# Immediate versus delayed integrated *point-of-care-ultrasonography* to manage acute dyspnea in the emergency department

**DOI:** 10.1186/2036-7902-6-5

**Published:** 2014-04-27

**Authors:** Concetta Pirozzi, Fabio G Numis, Antonio Pagano, Paolo Melillo, Roberto Copetti, Fernando Schiraldi

**Affiliations:** 1 Emergency Medicine Department, San Paolo Hospital, Via Terracina 219, Naples 80125, Italy; 2 Multidisciplinary Department of Medical Science, Second University of Naples, Via S. Pansini 5, Naples 80131, Italy; 3 Emergency Medicine Department, Latisana Hospital, Cassacco, Udine 33010, Italy

**Keywords:** Point-of-care-ultrasonography, Dyspnea, Diagnostic accuracy, Emergency Department

## Abstract

**Background:**

Dyspnea is one of the most frequent complaints in the Emergency Department. Thoracic ultrasound should help to differentiate cardiogenic from non-cardiogenic causes of dyspnea. We evaluated whether the diagnostic accuracy can be improved by adding a *point-of-care-ultrasonography* (*POC-US*) to routine exams and if an early use of this technique produces any advantage.

**Methods:**

One hundred sixty-eight patients were enrolled and randomized in two groups: Group 1 received an immediate POC-US in addition to routine laboratory and instrumental tests; group 2 received an ultrasound scan within 1 h from the admission to the Emergency Department. The concordance between *initial* and *final diagnosis* and the percentage of wrong diagnosis in the two groups were evaluated. Mortality, days of hospitalization in Emergency Medicine department and transfers to other wards were compared. Sensitivity and specificity of the routine protocol and the one including ultrasonography for the diagnosis of the causes of dyspnea were also analyzed.

**Results:**

Eighty-eight patients were randomized in group 1 and 80 in group 2. The concordance rate between initial and final diagnoses was significantly different (0.94 in group 1 vs. 0.22 in group 2, *p* < 0.005). The percentage of wrong *initial diagnosis* was 5% in group 1 and 50% in group 2 (*p* < 0.0001).

**Conclusions:**

Adding POC-US to routine exams improves the diagnostic accuracy of dyspnea and reduces errors in the Emergency Department.

## Background

Dyspnea is a subjective feeling of difficult, labored, or uncomfortable breathing and is among the most frequent complaints in patients admitted to the Emergency Department (ED). A rapid and correct diagnosis of the underlying cause is mandatory to provide early and targeted treatments. Discriminating between causes of dyspnea through clinical examination and initial routine approach, including history, electrocardiography (ECG) and laboratory results, may be inconclusive [1-3]. Chest radiograph (CXR) is currently the first routine imaging examination recommended for dyspneic patients: it is relatively easy to perform in ED and offers an immediate full-picture of the thorax. On the other side, CXR has several limitations. The major technical disadvantages are the limits of resolution, inability to visualize vascular structures and visualization of three-dimensional structures on a two-dimensional plane. Moreover, most of the times it is impossible to acquire posteroanterior and lateral projection in supine patients in the emergency setting; furthermore, ionizing radiation can cause damage to pregnant women. Finally, the report is not immediately available, and, above all, it has limited accuracy in detecting some diseases [4].

Another puzzling issue is that a valuable percentage of patients, almost elderly, show more than one cause of dyspnea [5,6].

Definitely, all these factors make the diagnostic approach challenging to dyspneic patients in ED.

Thoracic ultrasonography has been successfully introduced as a new and promising instrument for the diagnosis of different pulmonary diseases [7,8], and its ability to discriminate between cardiogenic and non-cardiogenic dyspnea is widely demonstrated in many valuable works [8-14].

However, thoracic ultrasonography alone could not correctly diagnose some causes of dyspnea and acute respiratory failure, such as pulmonary embolism and non-cardiogenic pulmonary edema (ARDS). In this last case, although some ultrasonography characteristics enable a clear distinction from cardiogenic edema [12], integrating the information derived from ultrasound assessment of the heart, may allow its complete definition and distinction.

The purpose of our study was to evaluate if a protocol including the standard diagnostic process (i.e. ABG, CXR, ECG, biochemical exams) and *point-of-care-ultrasonography* (*POC-US*) in ED would improve the accuracy of the emergency physician (EP) in the approach to acute undifferentiated dyspnea. Moreover, we attempted to explore if an immediate POC-US gives some advantages over a delayed ultrasonography evaluation of dyspneic patients. Our secondary purpose was to evaluate if the timing of the ultrasound could affect the number of dead, transferred or discharged patients and the days of hospitalization in the Emergency Medicine department.

## Methods

This was a single-center randomized prospective study about the use of POC-US in patients in ED presenting with undifferentiated dyspnea. All patients gave their informed consent to ultrasound examination (relatives consented for uncapable patients). The study was conducted in accordance to the principles of the declaration of Helsinki and approved by the Hospital Ethical Committee.

The study was performed between February and July 2012 in the ED (including also Emergency Medicine division) of the San Paolo Hospital, Napoli. We included 180 patients admitted to the ED (after pre-hospital care for some of them) because of complaining dyspnea, defined as either the sudden onset of shortness of breath without history of chronic symptoms or as increase in the severity of the chronic shortness of breath. Exclusion criteria were age <18 years, trauma, and ST elevation and myocardial ischemia.

The patients were enrolled only when one of the three investigators was present, although bedside ultrasonography is routinely performed in our ED by a consistent number of physicians.

The three participating investigators were two emergency residents and one attending physician, all well-trained in emergency ultrasonography.

**Figure 1:**
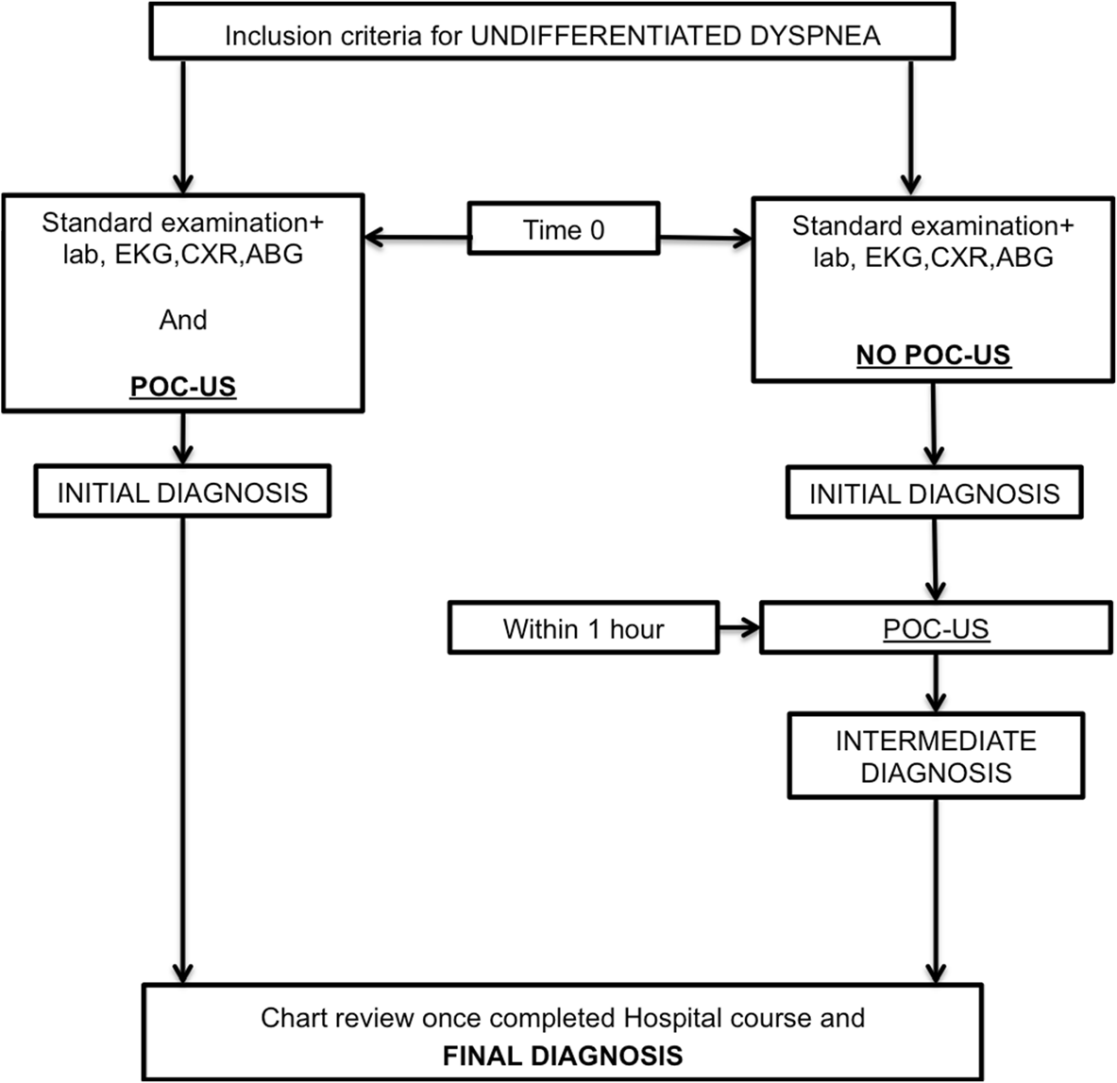
**Study design.** ECG, electrocardiogram; CXR, chest radiograph; ABG, arterial blood gases.

Patients were randomly assigned to group 1 or group 2. Group 1 received POC-US immediately, before any treatment (except for pre-hospital medications, if needed); group 2 followed the standard protocol of dyspnea and received POC-US within 60 min from the admission to the Emergency Department (Figure 1).

At time 0, medical history, medical treatment, signs and symptoms, 12-lead ECG, samples for standard blood tests (including troponin-T, D-dimers, WBC count and ultra-sensitive C-reactive protein) and blood gases (ABG) were taken from all patients, both in groups 1 and 2. CXR was obtained for all patients immediately after the physical examination and/or POC-US or after the initial treatment, as needed (although we do not have data on exact timing of CXR, we can assume that it was obtained between 15 and 20 min from the first medical contact).

In group 1, POC-US was performed only by one of the investigators.

POC-US exams were completed in the maximum of 8 min and were performed during nurse’s maneuvers. The physician-investigator was requested to indicate the *initial diagnosis*, derived from history and medical examination, ultrasonography, ABG, ECG and CXR result, within 30 min from fist medical contact (the adequate time to obtain CXR results).

In group 2, the physician who took in charge the patient was not a study member and made the initial diagnosis on the basis of history, medical examination, ABG, ECG and CXR result (within 30 min, as for group 1).

The POC-US in group 2 was performed for all patients by one of the three investigators as soon as one of the three was available, almost after the CXR execution, therefore likely between 20 to 60 min after the admission in the ED. In this last case, the investigator was blinded to the routine tests’ results before carrying out the ultrasound examination. After the POC-US, in group 2, the treating doctor revised the initial diagnosis and the therapeutic strategy, as necessary, according to ultrasonography findings, defining an *intermediate diagnosis*. The EPs were asked to indicate their initial diagnosis on a given form, choosing among six possibilities (Table 1), within 30 min from the first medical contact (an adequate time frame to obtain CXR results, given that the radiology is close to the emergency room). At the end of the hospital course, the patient’s charts were reviewed by two independent EPs, blinded both to ultrasonography findings and to the diagnostic hypothesis made by the investigators or the treating physicians. The *final diagnosis* was determined according to the standard criteria of each disease (Table 2) [15-20] by the two EPs, if they agreed independently on a diagnosis. If they disagreed, a third physician was consulted. The lists of possible initial, intermediate and final diagnoses coincide (Table 1).

**Table 1:** Causes of dyspnea and their occurrence

	**Number/percentage**
Acute heart failure (AHF)	64/38
Acute exacerbation of COPD (ECOPD)/asthma	53/31.5
Pneumonia	51/30.3
Acute respiratory distress syndrome (ARDS)	14/8.3
Massive pleural effusion	7/4.1
Acute pulmonary embolism	9/5.3

**Table 2:** Six possible causes of dyspnea and their diagnostic criteria

**Respiratory failure pattern**	**Diagnostic criteria**
AHF	Signs and symptoms of heart failure preserved or reduced systolic function of the left ventricle, CXR congestion
Acute exacerbation of COPD and asthma	History of COPD or asthma, typical findings at lung examination, airflow limitation, not fully reversible in COPD, fully reversible in asthma
Pneumonia	Fever, cough, leukocytosis, rales or abolished vesicular murmur, pulmonary infiltrate at CXR, positive cultures (eventually)
ARDS	Acute presentation within 1 week of a known clinical insult or new/worsening respiratory symptoms; chest imaging with bilateral opacities-not fully explained by effusions, lobar/lung collapse, or nodules; respiratory failure not fully explained by cardiac failure or fluid overload; PiO_2_/FiO_2_ < 200
Massive pleural effusion	Vesicular murmur abolished at lung auscultation and dullness at percussion, massive pleural effusion at CXR or US
Acute pulmonary embolism	Signs and symptoms, prediction rules indicating high probability; multidetector computed tomography positive for pulmonary embolism; dilated, hypokinetic right ventricle with pressure overload signs (when the embolism determines a significatively hemodynamic impairment)

When more than one cause of dyspnea was found in the same patient (mixed diagnosis), we considered as main cause of acute dyspnea/respiratory failure the pathological process that worsened the clinical situation (i.e. in a patient with AHF and pneumonia, although pneumonia could trigger the dyspnea, AHF is the clinical syndrome determining the respiratory failure).

The POC-US included lung, cardiac, inferior vena cava (IVC) and inferior limb vein compressive ultrasonography (CUS). A commercially available portable echography equipment (Mylab 30, Esaote Medical System, Genoa, Italy) was used to perform POC-US. Lung ultrasonography was performed with a 3.5 MHz convex probe, according to the eight-region technique [21]. A 2.5 to 3.5 MHz cardiac transducer was used in subcostal (SC) view for IVC evaluation and in SC and apical four-chamber (AP4) views for cardiac evaluation. Each cardiac exam was conducted using both views, except if one of them was not adequate. A qualitative eyeballing evaluation of the ejection fraction of the left ventricle (LV) was adopted [22].

A qualitative evaluation of the right ventricle (RV) dimension was used, considering as abnormal a RV/LV end diastolic diameter >0.9 in the AP4 view and >0.7 in the SC view [23], or a normal RV size as 60% of the LV size at the end diastole (that is a valid criterion both for AP4 and subcostal views). RV impaired function was estimated by reduction of contractility and movement of the RV free wall in the AP4 and SC views [24]; the absence of paradoxical interventricular septal motion was considered normal [24].

The IVC maximum diameter and the percentage of respiratory collapsibility (caval index) were recorded from SC view, 2 cm caudally from the junction of the right atrium, in order to estimate CVP: the combination of diameter >2 cm and excursion <50% was considered significant for a pressure >10 mmHg, while a diameter <2 cm together with an excursion >50% was diagnostic for a CVP <10 mmHg [23,25,26].

A 7.5-mHz linear probe was used for CUS of the groin and calf veins. All the ultrasonography images were saved on a hard disk.

The POC-US patterns used to define each clinical syndrome are resumed in Table 3.

**Table 3:** POC-US patterns and the corresponding combination of ultrasonography findings

**POC-US pattern**	**Signs**
AHF	Interstitial lung syndrome (the presence of multiple diffuse bilateral B-lines indicates interstitial syndrome) with reduced EF of the left ventricle or preserved EF with diastolic dysfunction
Pneumonia	Subpleural echo-poor region or one with tissue-like echo texture, with additional signs such as air/fluid bronchograms and adjacent B-lines (focal interstitial syndrome)
ARDS	Interstitial lung syndrome (the presence of multiple diffuse bilateral B-lines indicates interstitial syndrome), with non-homogeneous distribution of B-lines and spared areas, and normal systolic and diastolic function of the left ventricle
Massive pleural effusion	Anechoic space between parietal and visceral pleural and respiratory movement of the lung within the effusion
Possible pulmonary embolism	Dilated, hypokinetic RV with systolic septal dyskinesia plus dilated IVC with low collapsibility index (only found in massive or sub-massive pulmonary embolism) and eventually positive CUS of the groin or calf veins

The concordance between initial and final diagnosis in both groups 1 and 2 was analyzed by Cohen’s *κ* test.

The number of incorrect initial diagnosis in groups 1 and 2 was compared by Fisher’s test (*p* < 0.05 was considered significant).

Continuous variables were summarized as mean ± standard deviation and compared using the Student’s *t* test or Fisher’s test as adequate. Categorical variables were presented as frequencies and percentages and compared with × 2 tests. A *p* value of <0.05 was considered statistically significant. All calculations were performed with SPSS software, version 14.

A Student’s *t* test or a Fisher’s test, as adequate, was used for the comparison between the two groups in terms of days of hospitalization and deaths in the Emergency Medicine department, patients discharged or transferred to ICU and General Medicine wards.

## Results

One hundred sixty-eight patients made the final study sample; 88 were included in group 1, 80 in group 2. The two groups were well-matched for demographic and clinical characteristics (except for HR and RR which resulted significatively higher in group 2) and for previous medical history (clinical, demographic and laboratory characteristics showed in Table 4). All the POC-US exams were completed in the maximum of 8 min.

**Table 4:** Patients’ characteristics

	**G1**	**G2**	***P *** **value**
Age (years)	74.3 ± 11.4	74.5 ± 12.7	0.9
BMI (kg/m^2^)	28.5 ± 3	29 ± 3.4	0.3
Heart rate (bpm)	96.6 ± 20.8	110.6 ± 24	<0.05
Respiratory rate (bpm)	28.5 ± 5.8	30.4 ± 5	<0.05
SBP (mmHg)	148 ± 33	155 ± 37	0.19
DBP (mmHg)	85.6 ± 17.4	88 ± 18	0.38
pH	7.38 ± 0.1	7.37 ± 0.16	0.7
pCO_2_ (mmHg)	48.5 ± 17.5	44.1 ± 15.8	0.09
pO_2_ (mmHg)	63.8 ± 20.5	63.8 ± 28	1
FiO_2_ (%)	27.3 ± 11.6	25.6 ± 11.9	0.18
SpO_2_ (%)	87.3 ± 9.8	88 ± 8.4	0.6
PaO_2_/FiO_2_	255 ± 89	260 ± 87	0.7
HCO_3_^-^ (mmol/L)	27.4 ± 6.8	26 ± 6.2	0.16
Lactate (mmol/L)	2.03 ± 2.2	2.4 ± 1.9	0.24
Troponin-T (ng/mL)	0.15 ± 0.64	0.08 ± 0.15	0.34
CRP (mg/dL)	4.3 ± 7.3	6.8 ± 9.1	0.05
WBC (×10^3^/mL)	9.7 ± 4.8	10.6 ± 12	0.5
D-dimers (ng/dL)	1,175.3 ± 1,351	1,991 ± 1,039	0.9
COPD (%)	59	46	0.48
Renal failure (%)	23.8	22.5	0.83
Ischemic heart disease (%)	50	36	0.07
Heart failure (%)	22,7	20	0.76
Cerebral vasculopathy (%)	30	32.5	0.56
Diabetes (%)	25	27.5	0.7
Atrial fibrillation (%)	18	20	0.76

*BMI*, body mass index; *SBP*, systolic blood pressure; *DBP*, diastolic blood pressure; *CRP*, C-reactive protein; *WBC*, white blood cell; *COPD*, chronic obstructive pulmonary disease.

In group 2 half of the diagnoses changed after POC-US: the AHF was the most frequently changed diagnosis (25 out of 50, 50%); 48% of them were changed in pneumonia; 20% in ECOPD; 16% in ARDS; 12% in pulmonary embolism; 4% in massive pleural effusion. Among 23 initial diagnosis of ECOPD, 44% of them were modified after POC-US; 40% in pneumonia, 30% in AHF 30% in pulmonary embolism. Only one of the four initially diagnosed pneumonia was changed in AHF.

The Choen’s kappa between the initial (derived from standard exams and POC-US in group 1 and from standard exams alone in group 2) and final diagnosis was 0.94 (error 0.03; 95% confidence interval (CI) 0.88 to 1.00, *p* < 0.0001) in group 1, 0.22 (error 0.07; 95% CI 0.08 to 0.37; *p* = 0.006) in group 2.

The overall concordance between the diagnoses made after POC-US (initial diagnosis for group 1 and intermediate diagnosis for group 2) with final diagnoses was 0.95 (error 0.02; 95% CI 0.92 to 0.99, *p* < 0.0001). The frequency of incorrect initial diagnosis in group 1 was significantly lower than in group 2: 5% (4 out of 88) versus 50% (40 out of 80), (Fisher’s test *p* < 0.0001).

Six main ‘final’ diagnostic categories were selected; their occurrence in the total population is shown in Table 1.

We did not find spontaneous pneumothorax cases in our case series.

Thirty-one patients (18.4%) in the whole population had more than one diagnosis, but only the pathological process responsible for the precipitation of the clinical scenario was considered in the statistical analysis.

There was no significant difference in clinical outcomes included in the secondary purpose between the two groups (Fisher’s test *p* = 0.76 for deaths in Emergency Medicine, patients discharged or moved from Emergency Medicine to ICU or general medicine wards; the number of days in Emergency Medicine for patients in group 1 was 4.0 ± 2.0 days vs. 4.4 ± 2.1 days in group 2; *t* test *p* = 0.24).

We analyzed the sensitivity and specificity of the standard protocol and the standard protocol plus POC-US one for the diagnosis of each cause of dyspnea as reported in Table 5.

**Table 5:** Sensitivity and specificity of POC-US and standard protocol in diagnosis of acute dyspnea

	**After POC-US (G1 + G2)**^**a**^		**Standard protocol (G2)**^**a**^		**After POC-US (G2)**^**a**^	
**Diagnosis**	**Sensitivity (%)**	**Specificity (%)**	**Sensitivity (%)**	**Specificity (%)**	**Sensitivity (%)**	**Specificity (%)**
AHF	100	99	78.2	67.7	100	98.4
ECOPD/asthma	93	99	55.5	90.9	94.4	100
Pneumonia	92	98	14.2	97.1	93.3	98.5
ARDS	100	99	16.6	100	100	100
Massive pleural effusion	100	100	16.6	100	100	100
Acute pulmonary embolism	89	100	0	98.8	83.3	100

^a^ The diagnosis after POC-US is the initial diagnosis for group 1 (G1) and the intermediate diagnosis for group 2 (G2), the diagnosis after standard protocol is the initial diagnosis for G2. *AHF*, acute heart failure; *ECOPD*, exacerbation of chronic obstructive pulmonary disease; *ARDS*, acute respiratory distress syndrome.

## Discussion

This study tested the hypothesis that a POC-US protocol would increase the EP’s diagnostic accuracy in identifying the correct cause of symptomatic dyspnea in ED. If the ultrasound examination is performed at the same time of the visit in ED, the possibility of identifying the right cause of dyspnea is definitely higher than that given by the standard routine tests only. Although we could not find a difference in the clinical outcomes between the two groups of patients (immediate vs. delayed POC-US), the reduction of incorrect diagnosis after the first medical evaluation in the group receiving immediate POC-US justifies the use of ultrasound as soon as possible.

It was reported that lung ultrasound alone, or combined with N-terminal pro-BNP [8-14] and echocardiography [27], shows high diagnostic accuracy for differentiating cardiogenic from respiratory causes of dyspnea. Lichtenstein has proven the relevance of the BLUE protocol in the diagnosis of acute respiratory failure in ICU patients [28]. A recent study reports a higher accuracy of the lung-cardiac-inferior vena cava integrated ultrasound than the lung ultrasound alone or combined with BNP, only in discriminating AHF from pulmonary causes of dyspnea [29].

To our knowledge, there are few data in literature supporting the use of POC-US integrated with standard examination in dyspneic patients in ED not only to differentiate between AHF and ECOPD but also to rightly identify the pulmonary diseases potentially involved.

Our data confirm previous reports about the inadequacy of physical examination, CXR, ECG and ABG results alone in obtaining the right diagnosis in a given percentage of cases [6,30,31].

However, in our case series, the inaccuracy of initial diagnosis in group 2 derived from the traditional approach alone (before a delayed POC-US) is definitely lower than in other series. We think that the main reason of the low accuracy could be linked to the high percentage of elderly people with comorbidities among the subjects considered (mean age 74 years).

As previously reported, in elderly patients, cardiac and respiratory diseases frequently coexist; more than one acute cause of dyspnea is frequently found and moreover they usually have atypical clinical presentation, such as wheezing in AHF (cardiac asthma) or lack of infectious signs in pneumonia or non-respiratory symptoms in infectious disease [6]. Therefore, inappropriate initial treatment occurred in one third of the elderly people with acute respiratory failure, together with doubled in-hospital mortality, as compared to patients that received an appropriate treatment [6].

Even more, in these patients, a POC-US approach seems to be necessary to minimize diagnostic errors and to supply the best medical treatment.

Actually, our percentage of errors using the traditional approach to dyspnea was higher than the one described by Ray et al. [6] in elderly people.

There are, maybe, two other factors that could explain this gap between evidences and our results.

The first one is probably the poor quality of CXR in our center: the X-ray room is next to the emergency room, so there is only a little delay in performing the exam, but almost all the CXRs are obtained in anteroposterior projection only, in supine patients, because of difficulties in positioning them (elderly people are often non-collaborating and sometimes obese or neurologically compromised). So the quality of the exam is seriously affected and the sensitivity limited.

The second cause of such a low accuracy is maybe the lack of homogeneity among doctors in our ED, as regards to the specialty and the clinical experience and the lack of enough time, because of high flow of patients in the ED. But we could only speculate about and surely not eliminate these factors: selecting some physicians only to participate to the study could uniform medical skills, but should lead, of course, to a difficulty in obtaining an adequate sample of patients.

So we can say, only by assumption that the low accuracy of standard diagnostic approach reflects both the difficulties linked to elderly people management and the limits due to medical skills and lack of time in the emergency setting.

By the way, according to current literature, our results show that the sensitivity towards causes of dyspnea of the protocol including the POC-US is superior to the one with routine exams only. Previous studies showed that LUS has a better diagnostic performance than CXR in dyspneic and ICU patients [32,33], suggesting that lung ultrasound could replace CXR in ED. This last issue is also supported by a recent observational analysis, reporting that the clinical value of ‘on-demand’ individual CXRs was markedly higher than that of ‘daily routine’ CXRs, both for mechanically and non-mechanically ventilated patients [34].

Our study has surely some limitations. First of all, this is a single-center study, with a small number of patients, enrolled by only three EPs trained in ultrasonography. Because of the small sample size, some causes of dyspnea resulted in low recurrence, limiting the reproducibility of data relative to the ability of ultrasound in detecting them.

Secondly, we could not find in our population a benefit of immediate versus delayed POC-US on clinical outcomes, such as in-hospital mortality.

Thirdly, some may argue that performing ultrasonography after an initial treatment could influence some ultrasonography pathologic findings, because of their resolution. This should be true for some cases of cardiogenic pulmonary edema, although it is described that almost 2 h are necessary to appreciate B-lines resolution after medical or continuous positive airway pressure (CPAP) therapy [35,36].

Fourthly, the ultrasound protocol used in this study requires knowledge of cardiac, thoracic and veins ultrasonography. This surely requires a special training, but it is elsewhere described that each single component of this protocol can also be performed by emergency physicians with limited training [37,38].

Despite these limitations, we believe that this study strongly supports the routine use of a POC-US protocol, including examination of lung, heart, inferior vena cava and lower limb veins, together with routine exams, as soon as patients come to ED complaining of dyspnea.

Additional research on larger populations could be useful to evaluate the relationships between timing of POC-US in dyspnea in ED and patient’s main outcomes, and the impact on dyspnea associated costs, given the ability of early integrated ultrasound to reduce the need of additional, more sophisticated tests, in some challenging clinical scenarios.

## Conclusions

Adding POC-US to routine exams can really improve diagnostic accuracy in patients complaining of dyspnea and can reduce errors in the ED, although there is no benefit of immediate versus delayed POC-US on clinical outcomes.

## Abbreviations

ABG: arterial blood gases; AHF: acute heart failure; AP4: apical 4 chamber; ARDS: acute respiratory distress syndrome; CUS: compressive ultrasonography; CXR: chest x-ray; ECG: electrocardiography; ECOPD: exacerbation of chronic obstructive pulmonary disease; ED: Emergency Department; EP: emergency physician; IVC: inferior vena cava; LV: left ventricle; POC-US: point-of-care ultrasound; RV: right ventricle; SC: subcostal.

## Competing interest

The authors declare that they have no competing interests.

## Authors’ contributions

CP conceived the study, selected patients, performed ultrasound, supervised the data collection and drafted the manuscript. FN conceived the study, selected patients, performed ultrasound, approved and revised the manuscript. AP selected patients, performed ultrasound, supervised data collection, approved and revised the manuscript. FS and RC managed the data, including quality control, contributed substantially to manuscript revision. PM provided statistical advice on study design and analyzed the data. All authors read and approved the final manuscript.
